# Extensive malignant melanoma of the oral cavity: a rare occurrence

**DOI:** 10.4322/acr.2021.299

**Published:** 2021-08-20

**Authors:** Pradeep Pradhan, Amit Kumar Adhya

**Affiliations:** 1 All India Institute of Medical Sciences, Department of ENT and Head Neck Surgery, Bhubaneswar, Odish, India; 2 All India Institute of Medical Sciences, Department of Pathology, Bhubaneswar, Odish, India

**Keywords:** Malignant melanoma, Oral cavity, Management

## Abstract

Primary malignant melanoma of the oral cavity is a rare tumor in clinical practice. Extensive malignant melanomas are still very rare in the literature. Patients with malignant melanoma of oral cavity are often diagnosed at the advanced stage of the disease due to their painless and nonspecific radiological findings. Histopathology is the gold standard for the final diagnosis and staging of the tumor. Surgery followed by radiotherapy is the standard treatment offered to patients with malignant melanoma. Here we present a rare case of extensive malignant melanoma of oral cavity which was successfully managed by surgical excision followed by adjuvant radiotherapy.

## INTRODUCTION

Malignant melanoma is an epithelial cell tumor resulting from the malignant transformation of melanocytes or melanocytes precursors in the skin.^1^ It rarely involves the mucous membrane. It was first described by Weber in 1859 and was later named as “melanotic sarcoma” by Lucke in 1869, as quoted by Pandey.^2^ Malignant melanoma of the oral cavity accounts for approximately 0.2–8% of all melanomas and has a poorer prognosis compared to the dermatological counterpart.^3^ In addition, it has a higher tendency for early metastasis to the regional lymph nodes and distant metastasis to the lung and liver.^4^ This disease is more prevalent in males in their fifth decades of life, and the male to female prevalence ratio is 2:1.^5^ The hard palate and maxillary gingiva^6^ are commonly involved in the disease, and the involvement of lower and upper alveoli and mandibular gingiva is extremely rare.^7^ The clinical presentation of malignant mucosal melanoma is variable in contrast to the carcinoma and often results in considerable delay in medical consultation due to the absence of pain in patients.^8^^,^ 9 Surgical excision of the tumor with a wider margin of resection (3.0 cm) is considered as the primary treatment modality for mucosal malignant, although adjuvant radiotherapy is warranted in some cases of extensive lesion with regional metastasis. Here, we describe a case of extensive malignant melanoma of retromolar trigone affecting both upper and lower alveoli, the buccal mucosa, hard palate, and soft palate. This case was successfully managed with wide local excision and segmental mandibulectomy, along with infrastructural maxillectomy where the defect was successfully reconstructed with pectoralis major myocutaneous flap.

## CASE REPORT

**Figure 1:**
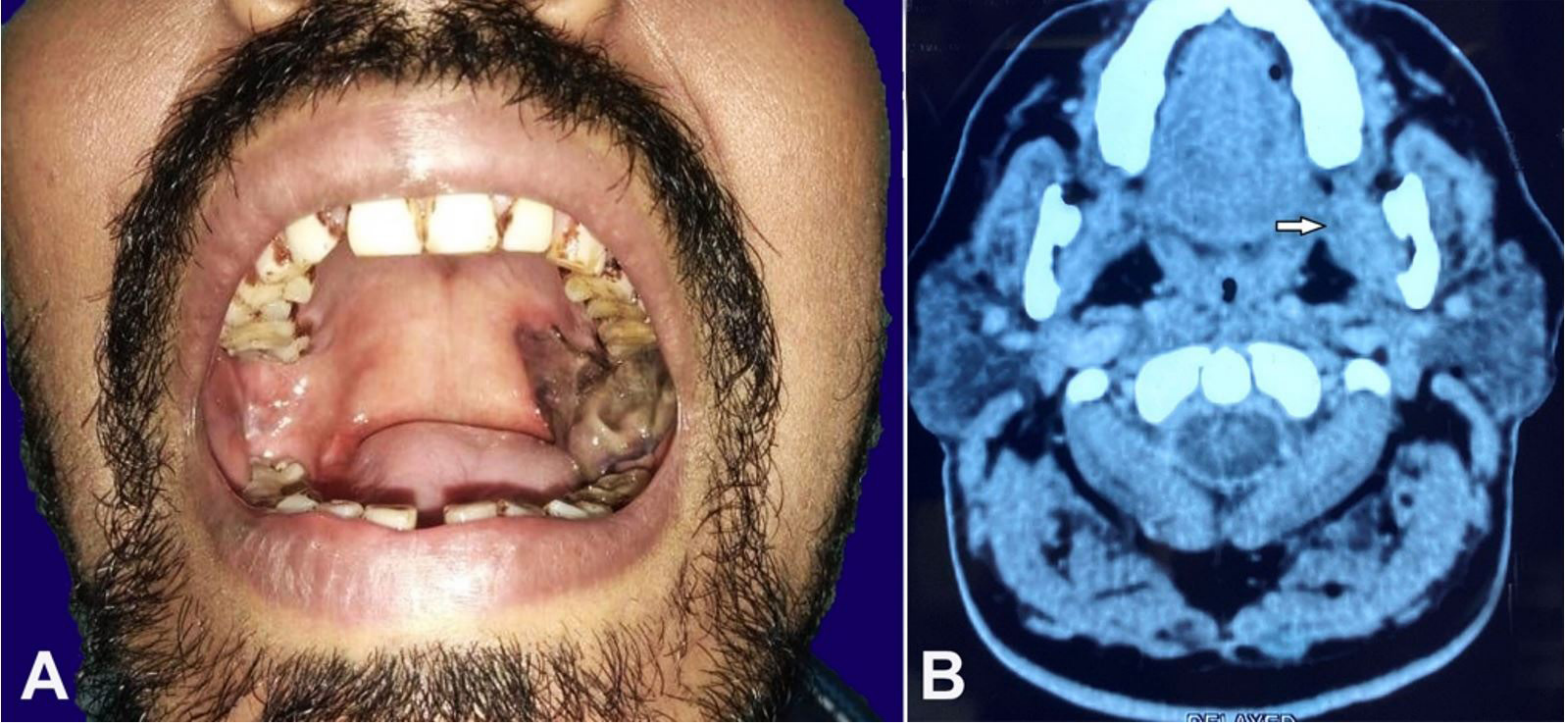
**A –** Tumor was found to involve the retromolar trigon (Left), extending laterally to the buccal mucosa and gingivobuccal sulcus and superiorly to the alveolar process and the hard and soft palates; **B –** A contrast-enhanced CT scan (Axial section) revealed a soft tissue mass in the left RMT, involving the body and ramus of mandible (left side) and the upper alveolus.

A 35-year-old male presented to the Department of Otorhinolaryngology with a pigmented mass over the left retromolar trigone that existed for six months. The patient had a history of occasional paroral bleeding for the past three months and loosening of the upper last molar tooth for two months. He had consulted with a dentist for the same and was advised topical medication for two months. He was then referred to the Otorhinolaryngologist as the symptoms failed to respond to the treatment. Physical examination revealed a pigmented proliferative mass involving the gingival surface of the retromolar trigon, extending laterally to the buccal mucosa and gingivobuccal sulcus and superiorly to the alveolar process of the maxilla (Figure 1A). No palpable cervical lymphadenopathy was detected in the patient. A contrast-enhanced computed tomography (CECT) scan revealed a soft tissue mass in the left retromolar trigone (RMT) space. This mass involved the maxillary alveolus and palate without erosion of the palatal bone (Figure 1B).

**Figure 2:**
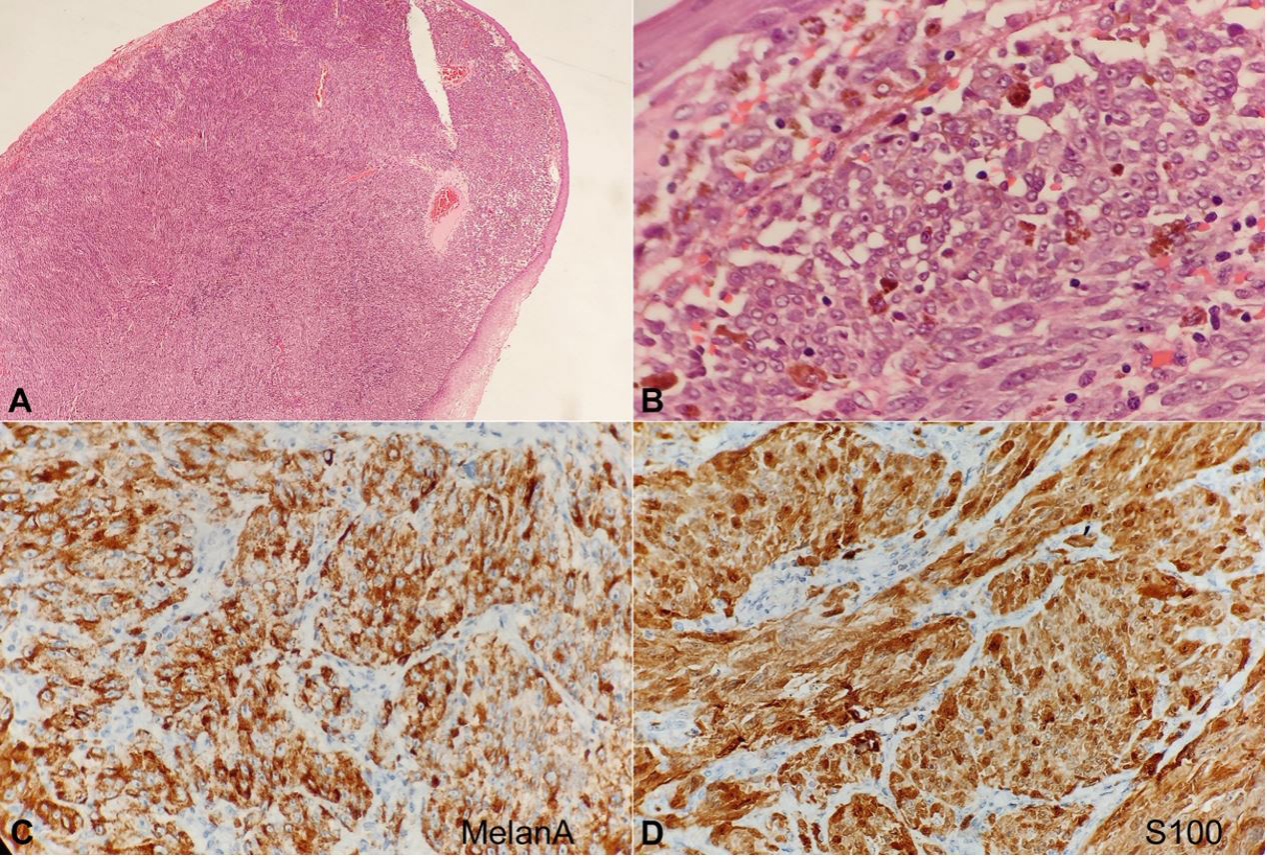
Photomicrographs of the tumor. **A –** Section showing intact squamous epithelium and diffuse infiltration of the subepithelium by tumor cells (H&E, 40x); **B –** High power view showing round and spindle cells with prominent nucleoli, and melanin in the cells (H&E, 400x); **C –** Immunohistochemistry for Melanin A showing strong positivity in the tumor cells (200x); **D –** Immunohistochemistry for S-100 showing strong positivity in tumor cells (400x).

All routine hematological and biochemical investigations were found to be normal. The incisional biopsy of the lesion indicated intact squamous epithelium and diffuse infiltration of the subepithelium by spindle-shaped tumor cells with prominent nucleoli. Immunohistochemistry showed strong positivity for Melanin A and S-100 in the tumor cells (Figure 2A,B,C,D).

**Figure 3:**
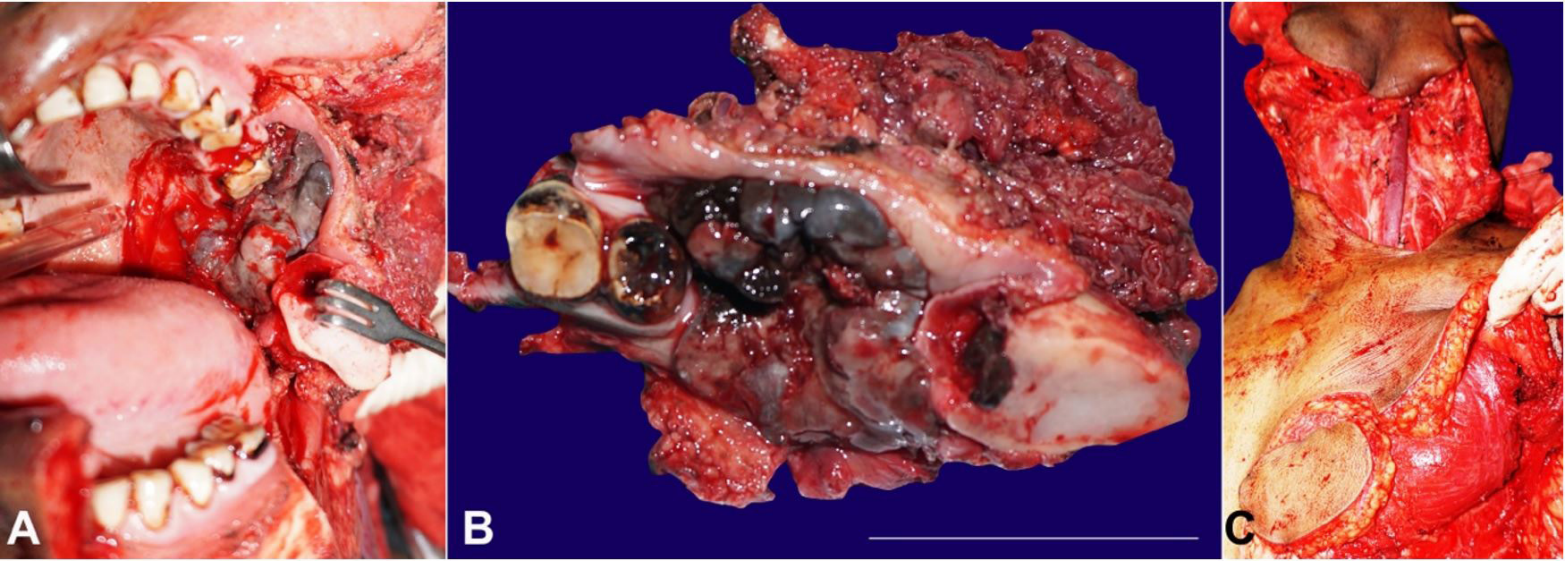
**A –** Intraoperative view showing wide local excision of the tumor along with left segmental mandibulectomy and infrastructural maxillectomy; **B –** Surgical specimen after the excision of the tumor; **C –** Harvation of the pectoralis major myocutaneous flap (left side) for soft tissue reconstruction.

The CECT scan of the whole abdomen and chest was found to be normal. Written informed consent of the patient were taken for surgical excision after the nature of the lesion, treatment options, prognosis of the disease, and the possibility of the need for adjuvant chemoradiation was explained to him and his family. Wide local excision along with left segmental mandibulectomy and infrastructural maxillectomy was performed (Figure 3A). The tumor was 5.6 × 3.5 × 4.9 cm in size and weighed 62.2 g (Figure 3B). The defect was reconstructed with pectoralis major myocutaneous flap, spreading from the palatal margin to the floor of the mouth (Figure 3C).

**Figure 4:**
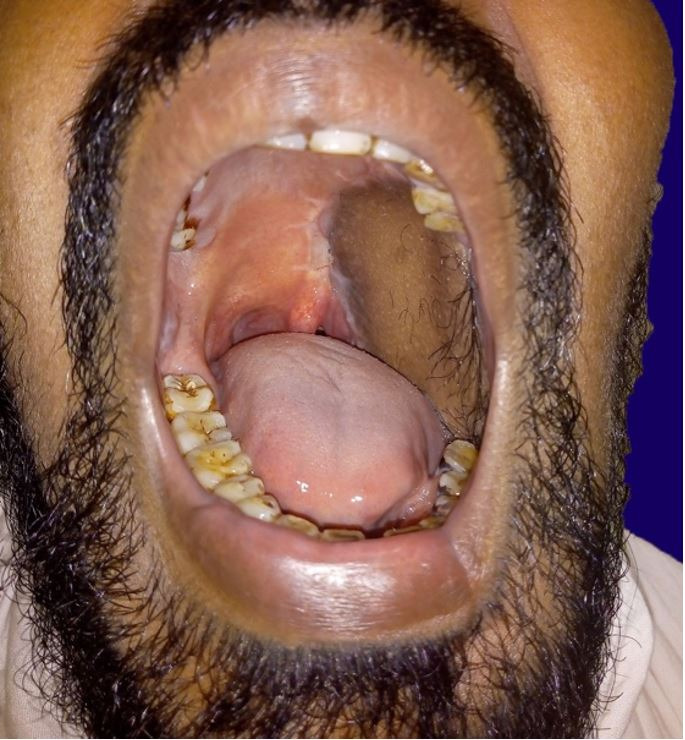
Complete closure of the surgical defect with pectoralis major myocutaneous flap six weeks after the surgery.

The patient was started on Ryle's tube feeding 24 hours after the surgery and was discharged seven days after the surgery. Histopathological section was suggestive of malignant melanoma with tumor-free resected margin with absence of lymphatic/ bony involvement. The patient was advised for postoperative radiotherapy, considering the grave prognosis of the disease. He has been on regular follow-up for the past 24 months and found asymptomatic without the recurrence of the disease (Figure 4).

## DISCUSSION

Primary malignant melanoma of oral mucosa is a rare tumor to encounter in clinical practice and accounts for about 0.2–8% of all melanomas.^3^ Although the hard palate and the maxillary gingiva^6^ are commonly involved in the disease, the involvement of both upper and lower alveoli, hard palate and soft palate mucosa is extremely rare.^7^ No definitive predisposing factors for malignant mucosal melanoma have been identified yet, but cigarette smoking, dental trauma, alcohol intake, and immune suppression may be closely associated with disease incidence.^10^ In the present case, we did not find any association of the predisposing factors in the patient, which is supported by previous literature describing the de novo origin of the tumor.^11^ It is predominantly found in males (male: female, 2:1), and patients in their fifth decade of life are preferably affected by the disease.^5^ In the early stage of the tumor, most of the patients are asymptomatic, and very few patients can have pain, bleeding, or ulcerated lesions depending on the size and extension of the lesion.^2^ In the present case, the patient had a history of peroral bleeding during chewing without any other significant complaints. Malignant melanoma is diagnosed mainly by histopathological examination, which reveals spindle to oval cells with a higher degree of cellular atypia, abundant eosinophilic cytoplasm, large nuclei, and prominent eosinophil nucleoli**.** More than 95% of the lesions are anti-S-100 antigen-positive, and more specific markers include HMB45, Melan-A, and anti-tyrosinase.^12^ Mucosal melanoma of the oral cavity is classified as *in situ* oral mucosal melanoma, invasive oral mucosal melanoma, and mixed *in situ* invasive lesions.^13^ Our case had invasive lesion. Greene et al. proposed three criteria for the diagnosis of primary malignant melanoma of oral cavity: the presence of malignant melanoma of oral cavity, exclusion of melanoma in any primary sites and histopathological confirmation of the disease.^14^ In most cases, malignant melanoma is managed by surgery taking a generously healthy mucosal margin, as described by Zitelli et al.^15^ The safety margin should be at least 1.5 cm for head and neck melanomas and 2.5 cm for melanomas larger than 3 cm in diameter. In the present case where wide local excision was done, the healthy mucosal margin was considered to be approximately 3.0 cm, along with left segmental mandibulectomy and upper alveolectomy. The defect was reconstructed with pectoralis major myocutaneous flap, spreading from the palatal margin to the floor of the mouth. The disease had poor prognosis, and the five-year survival rate is 5% to 20%.^16^ This could be because of the indolent nature of the lesion and late stage of presentation, as demonstrated in the present case. Moreover, due to the hidden location and rich vascularization of the oral mucosa, 75% of patients present with lymphatic metastasis and 50% of them present with distance metastasis, especially to the liver and lung.^17^ Hence, clinical- histopathological staging is a useful prognostic factor in the management of the disease.^18^^,^ 19

Stage I: The presence of the primary tumor (T_any_N_0_M_0_)

Level I: Pure*in situ*melanoma with either absence of invasion or*in situ* melanoma with "microinvasion."

Level II: Involvement of the lamina propria

Level III: Invasion into the deep skeletal tissue (skeletal muscle, bone, or cartilage).

Stage II: Metastasis of tumor to regional lymph nodes (T_any_N_1_M_0_)

Stage III: Metastasis of tumor to distant sites (T_any_N_any_M_1_).

Based on the above clinical staging, the tumor was considered to be stage I (Level III) as it invaded the mandibular bone. Although surgical excision is considered a curative option for early lesions of malignant melanoma, patients with extensive disease can be subjected to adjuvant chemoradiation/immunotherapy, despite poor radiosensitive tumor.^20^ In the present case, the patient was subjected to adjuvant radiotherapy due to extensive disease, although we did not get any locoregional/systemic metastasis in the final histopathological report. In recent years, immune therapy has been considered for better survival of patients with malignant mucosal melanoma.^21^ In the present case, the patient is doing well after adjuvant radiotherapy with good cosmesis and without any recurrence.

## CONCLUSION

Primary malignant melanoma of the oral cavity is a rare tumor in clinical practice. An extensive lesion with the involvement of retromolar trigon, superior and inferior alveolar mucosa, buccal mucosa, and palates is extremely rare and yet to be described in the literature. As these lesions are painless with nonspecific radiological features, patients are often diagnosed at advanced stage of the disease. Although the tumor possesses a poor prognosis, standard treatment with surgery and adjuvant radiotherapy can provide a favorable outcome with better survival of a patient.

## References

[1] Sabanathan S, Eng J, Pradhan GN (1989). Primary malignant melanoma of the esophagus. Am J Gastroenterol.

[2] Pandey M, Mathew A, Iype EM, Sebastian P, Abraham EK, Nair KM (2002). Primary malignant mucosal melanoma of the head and neck region: pooled analysis of 60 published cases from India and review of literature. Eur J Cancer Prev.

[3] Ebenezer J (2006). Malignant melanoma of the oral cavity. Indian J Dent Res.

[4] Tas F, Keskin S (2013). Mucosal melanoma in the head and neck region: different clinical features and same outcome to cutaneous melanoma. ISRN Dermatol.

[5] Strauss JE, Strauss SI (1994). Oral malignant melanoma: a case report and review of literature. J Oral Maxillofac Surg.

[6] Hashemi Pour MS (2008). Malignant melanoma of the oral cavity: a review of literature. Indian J Dent Res.

[7] Hayashi T, Ito J, Katsura K (2002). Malignant melanoma of the mandibular gingiva; the usefulness of fat saturated MRI. Dentomaxillofac Radiol.

[8] Smyth AG, Ward-Booth RP, Avery BS, To EW (1993). Malignant melanoma of the oral cavity- an increasing clinical diagnosis?. Br J Oral Maxillofac Surg.

[9] Clark DB, Priddy RW, Kaburda M (1989). Primary malignant melanoma of the maxillary sinus: case report and literature review. J Oral Maxillofac Surg.

[10] Rapidis AD, Apostolidis C, Vilos G, Valsamis S (2003). Primarymalignant melanoma of the oral mucosa. J Oral Maxillofac Surg.

[11] Colllins B, LeonBarnes E, Abernethy J (2005). Oral Malignant melanoma. J Clin Oncol.

[12] Femiano F, Lanza A, Buonaiuto C, Gombos F, Di Spirito F, Cirillo N (2008). Oral malignant melanoma: a review of the literature. J Oral Pathol Med.

[13] Müller S (2010). Melanin-associated pigmented lesions of the oral mucosa: presentation, differential diagnosis, and treatment. Dermatol Ther.

[14] Ebenezer J (2006). Malignant melanoma of the oral cavity. Indian J Dent Res.

[15] Zitelli JA, Brown CD, Hanusa BH (1997). Surgical margins for excision of primary cutaneous melanoma. J Am Acad Dermatol.

[16] Bouchareb N, Souldi H, Rouadi S (2014). Malignant melanoma of the mandibular gingiva. Rev Laryngol Otol Rhinol (Bord).

[17] Wong JH, Cagle LA, Storm FK, Morton DL (1987). Natural history of surgically treated mucosal melanoma. Am J Surg.

[18] Sharma N (2012). Primary oral malignant melanoma: two case reports and review of literature. Case Rep Dent.

[19] Sivapathasundharam B (2016). Shafer’s text book of oral pathology.

[20] Hansson J (1997). Systemic therapy of malignant melanoma. Med Oncol.

[21] Zitelli JA, Brown CD, Hanusa BH (1997). Surgical margins for excision of primary cutaneous melanoma. J Am Acad Dermatol.

